# Elranatamab for Relapsed/Refractory Multiple Myeloma With Severe Renal Impairment Requiring Hemodialysis

**DOI:** 10.1002/hon.70120

**Published:** 2025-07-20

**Authors:** Michèle Hoffmann, Barbara Jeker, Uyen Huynh‐Do, Yara Banz, Jeanne Godau, Elisabeth Weber, Ulrike Bacher, Thomas Pabst

**Affiliations:** ^1^ Department of Medical Oncology Inselspital Bern Bern University Hospital University of Bern Bern Switzerland; ^2^ Division of Nephrology and Hypertension Inselspital Bern University Hospital University of Bern Bern Switzerland; ^3^ Institute of Tissue Medicine and Pathology University of Bern Bern Switzerland; ^4^ Department of Medical Oncology & Hematology Spital STS Thun Thun Switzerland; ^5^ Department of Nephrology Spital STS Thun Thun Switzerland; ^6^ Department of Hematology and Central Laboratory Inselspital Bern Bern University Hospital University of Bern Bern Switzerland

**Keywords:** bispecific antibody, elranatamab, hemodialysis, hypogammaglobulinemia, myeloma, relapse

## Abstract

Relapsed/refractory multiple myeloma (RRMM) patients with dialysis‐dependent renal impairment face limited therapeutic options due to exclusion from clinical trials, a lack of evidence‐based guidelines, and inferior outcomes. Bispecific antibodies targeting B‐cell maturation antigen (BCMA) have shown promise in RRMM treatment but remain understudied in this vulnerable population. To illustrate this issue, we introduce the case of a 68‐year‐old female with triple‐class RRMM and end‐stage renal disease requiring hemodialysis, treated with elranatamab as a second line treatment following progression after therapy with daratumumab, bortezomib, lenalidomide, and dexamethasone. Despite experiencing grade I cytokine release syndrome during the initial administrations, symptoms were managed effectively with tocilizumab and dexamethasone, allowing treatment continuation. The patient achieved a very good partial remission within 7 weeks. Although hemodialysis dependence persisted, the therapy was well‐tolerated with manageable adverse events. According to the literature, BCMA‐directed immunotherapies, including teclistamab, belantamab mafodotin, and idecabtagene vicleucel, have shown efficacy in dialysis‐dependent RRMM patients, though data remain limited. Pharmacokinetic analyses indicate that mild or moderate renal impairment does not have a significant impact on the pharmacokinetics of elranatamab. Although no retrospective studies or case series have investigated the use of elranatamab in dialysis‐dependent patients, a single case report suggests that its administration is both feasible and well‐tolerated in this population despite the absence of comprehensive pharmacokinetic data. This review highlights feasibility, safety, and encouraging efficacy of elranatamab in managing RRMM in a dialysis‐dependent patient, representing the second case report in the literature. By providing real‐world evidence for the use of bispecific antibodies in end stage renal disease patients, this review emphasizes the potential for expanding therapeutic options to this vulnerable population while highlighting the need for vigilant monitoring of infection prevention and management. Prospective studies are warranted to validate these findings and optimize therapeutic strategies for patients with RRMM and severe renal impairment.

## 1 Introduction

Relapsed/refractory multiple myeloma (RRMM) provides a number of significant therapeutic challenges, particularly in patients with severe renal impairment requiring hemodialysis. While renal dysfunction is a common complication of multiple myeloma affecting up to 50% of patients at diagnosis, only 2%–5% ultimately progress to dialysis dependency. These patients face inferior clinical outcomes and are usually excluded from clinical trials, resulting in limited evidence to guide treatment strategies.

Bispecific antibodies targeting the B‐cell maturation antigen (BCMA), such as elranatamab and teclistamab, have emerged as promising agents in the treatment of RRMM [1, 2, 3]. While simultaneously engaging BCMA on malignant plasma cells and CD3 on T cells, these therapies potentiate immune‐mediated cytotoxicity. Elranatamab has demonstrated robust efficacy in heavily pretreated RRMM patients, with an overall response rate of 61% in the Phase 2 MagnetisMM‐3 study [1]. However, registration trials have excluded patients with severe renal impairment or those requiring dialysis, leaving a critical gap in our knowledge on safety and efficacy in this subgroup.

In this article, we present an illustrative patient case of a 68‐year‐old female diagnosed with high‐risk multiple myeloma characterized by high risk cytogenetic abnormalities as well as end‐stage renal disease (ESRD) requiring dialysis. The patient initially achieved a very good partial response (VGPR) with a quadruplet regimen of daratumumab, bortezomib, lenalidomide, and dexamethasone, although early disease progression occurred approximately 1 year later. Second‐line treatment with the BCMA‐targeting bispecific antibody elranatamab was initiated, complicated by grade I cytokine release syndrome (CRS) and subsequent cutaneous toxicity, which were managed successfully without treatment delays. Remarkably, the patient achieved complete cytogenetic remission and minimal residual disease (MRD) negativity, underscoring the therapeutic potential of elranatamab, albeit with persistent dialysis dependence and immunosuppression‐related complications.

Diligent research of available literature revealed that pharmacokinetic analyses suggest mild to moderate renal impairment has minimal impact on elranatamab metabolism [1]. However, data on patients with ESRD requiring dialysis are lacking. Notably, Van de Wyngaert et al. [4] presented a case report describing successful elranatamab administration in a dialysis‐dependent patient who achieved a complete response within 1 month of therapy. In contrast, more data are available for teclistamab in patients with severe renal impairment. A French retrospective study involving 15 dialysis‐dependent RRMM patients demonstrated promising outcomes with teclistamab, with 93% of patients responding to treatment [5]. Another case series from the United States also confirmed the feasibility of teclistamab in four dialysis‐dependent patients [6].

Thus, this second case report in literature and review highlight feasibility, safety, and remarkable efficacy of elranatamab and teclistamab in patients with RRMM who are dialysis‐dependent, addressing key challenges such as CRS, infection risk, and pharmacokinetic considerations related to hemodialysis. Notably, only a single case report on the use of elranatamab in a dialysis‐dependent RRMM patient has been published to date [4], underscoring the pioneering nature of this therapeutic approach.

### 1.1 Patient Case Report

A 68‐year‐old female patient was diagnosed with triple‐class refractory, Revised International Staging System (R‐ISS) stage III multiple myeloma and dialysis‐dependent renal failure, in May 2023. Initial diagnostic work‐up identified significant proteinuria (4.8 g/24 h), acute kidney injury with a creatinine level of 486 μmol/L, hypercalcemia (3.32 mmol/L), and anemia (hemoglobin 75 g/L). Beta‐2‐microglobulin was markedly elevated at 23.62 mg/L, LDH levels were slightly elevated (379 U/L). A IgG kappa paraprotein level of 35.8 g/L was found. Levels of free light chain (FLC) kappa were markedly elevated (FLC kappa: 8067 mg/L; FLC lambda: 3.33 mg/L; ratio: 2423). Bone marrow infiltration by plasma cells was 80%, characterized by an immunophenotype of Ig kappa light chain restriction, BCMA+, Cyclin D1+, CD56‐, CD20‐, and c‐kit‐, without evidence of amyloid deposition. Cytogenetic analysis (array CGH) identified a high‐risk karyotype, including deletion of 17p13.1 (TP53), gain of 1q21, translocation t (11; 14), del16q, hypodiploidy (loss of the entire X chromosome and parts of chromosomes 10 and 16), and a ploidy profile (approximately 90% ±2n, 10% +4n). A whole‐body CT scan revealed multiple osteolytic bone lesions.

**FIGURE 1:**
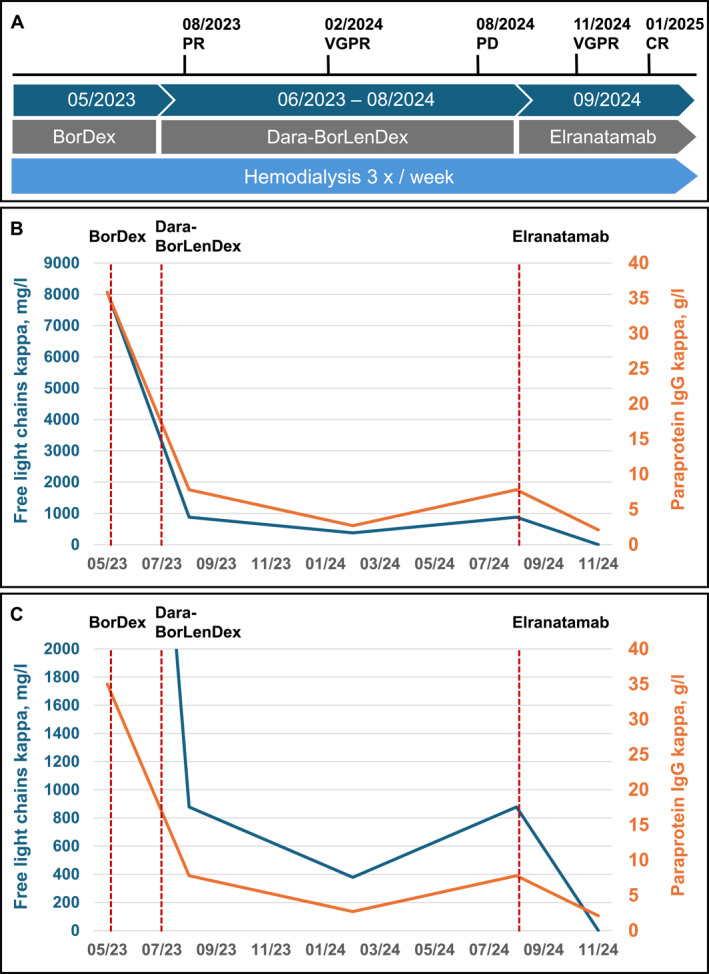
Timeline composite figure indicating disease course and corresponding treatments (A), and the dynamic of myeloma parameters: serum free light chain kappa (blue) and paraprotein IgG kappa (orange) levels. The first red vertical line indicates the time point of initiation of the first BorDex cycle, the second line the time point of initiation of the therapy with Dara‐BorLenDex, and the third line the time point of initiation of the treatment with Elranatamab. (B and C). The free light chains kappa and the paraprotein IgG kappa were measured prior to each dialysis session. BorDex: Bortezomib, Dexamethasone; Dara‐BorLenDex: Daratumumab, Bortezomib, Lenalidomide, Dexamethasone; CR: complete response; PR: Partial response; VGPR: Very good partial response; PD: Progressive disease.

All CRAB criteria (hypercalcemia, renal failure, anemia, osteolytic bone lesions) were fulfilled, necessitating the initiation of an emergency treatment regimen with bortezomib and dexamethasone. Simultaneously, hemodialysis was started for anuric kidney failure, scheduled three times per week. The disease evolution and corresponding treatments are outlined in Figure 1. A combination of daratumumab, bortezomib, lenalidomide, and dexamethasone was initiated as first‐line therapy. The early clinical course following starting therapy was complicated by pneumonia due to parainfluenza and a bacterial superinfection, hypervolemia, and cardiac decompensation, necessitating admission to the intensive care unit and intubation with mechanical ventilation for three consecutive days. Due to persistent anuric renal failure, dialysis was continued at a frequency of three times per week.

The patient achieved a VGPR in February 2024, representing the best treatment response after 11 consecutive cycles of therapy (with minimal FLC kappa: 212 mg/L, monoclonal IgG kappa paraprotein: 2.7 g/L). The extended first‐line regimen consisting of daratumumab, bortezomib, lenalidomide, and dexamethasone was well tolerated, particularly without significant polyneuropathy. With respect to the potential reversibility of dialysis‐dependent renal failure, a melphalan‐based high‐dose consolidation chemotherapy (dosis melphalan 140–200 mg/m^2^) with autologous stem cell transplantation (HDCT/ASCT) was deferred at first remission [7, 8, 9].

Since July 2024, thus 15 months after first diagnosis, a continuous increase in myeloma markers was observed (FLC kappa levels rising to 844 mg/L and IgG kappa paraprotein levels increasing to 7.8 g/L). This trend was confirmed during follow‐up, indicating that approximately 1 year after initiating first‐line therapy with daratumumab, bortezomib, lenalidomide, and dexamethasone, the patient presented with progressive disease under the quadruplet regimen. A whole‐body CT scan revealed no new osteolytic bone lesions. The early progression likely mirrored the presence of the two adverse prognostic markers del (17p) and gain of 1q21.

Given the situation of a triple‐refractory myeloma and ongoing dialysis‐dependent renal failure, a second‐line therapy with the anti‐BCMAxCD3 bispecific antibody elranatamab was initiated in September 2024. Infectious prophylaxis included sulfamethoxazole/trimethoprim 0.5 tablets three times weekly against Pneumocystis jirovecii (PcP), and valacyclovir 250 mg twice daily against varicella‐zoster virus, with dosage adjustments for renal function.

**FIGURE 2:**
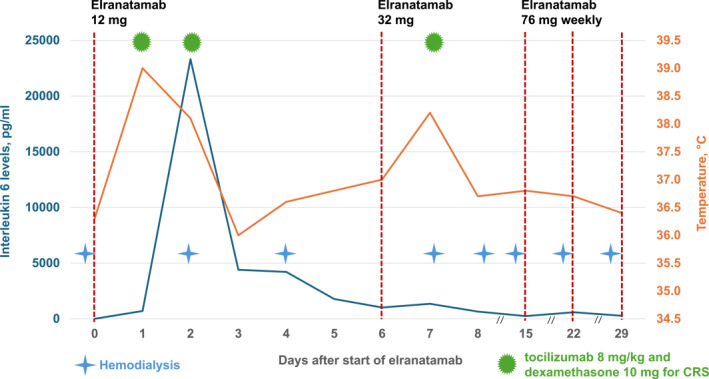
Dynamics of interlukin 6 levels (blue) and the course of temperature (orange). The red vertical lines indicate the timepoint of elranatamab therapy. The blue stars indicate the dialysis sessions that took place prior to the administration of elranatamab. The green dots represent cytokine release syndrome (CRS) along with the corresponding treatment consisting of tocilizumab at 8 mg/kg body weight and dexamethasone 10 mg intravenously.

Elranatamab treatment started with a standard ramp‐up phase following premedication with dexamethasone, levocetirizine dihydrochloride 5 mg, and paracetamol. Initial step‐up dosing was at 12 and 32 mg before continuing with 76 mg weekly, administered post‐dialysis. The patient developed grade I CRS twice after the first dose, presenting with fever up to 39.0°C, generalized myalgias, chest pain, and an IL‐6 peak of 23,307 pg/mL. Acute coronary syndrome was excluded. Tocilizumab (8 mg/kg) and dexamethasone (10 mg) were administered twice following the first dose of elranatamab, leading to complete CRS resolution and IL‐6 reduction. The second elranatamab dose was given 48 h post‐CRS resolution, precipitating grade I CRS recurrence with symptom relief despite a peak fever of 38.2°C. A third dose of tocilizumab (8 mg/kg) and dexamethasone (10 mg) led to complete resolution of the fever and to a decline in IL‐6 levels, which had peaked at 1348 pg/ml (Figure 2). On day 15, after CRS resolution, a third elranatamab dose (76 mg) was administered, maintaining dexamethasone premedication. The patient experienced no further CRS, macrophage activation syndrome or immune effector cell‐associated neurotoxicity syndrome (ICANS).

**FIGURE 3:**
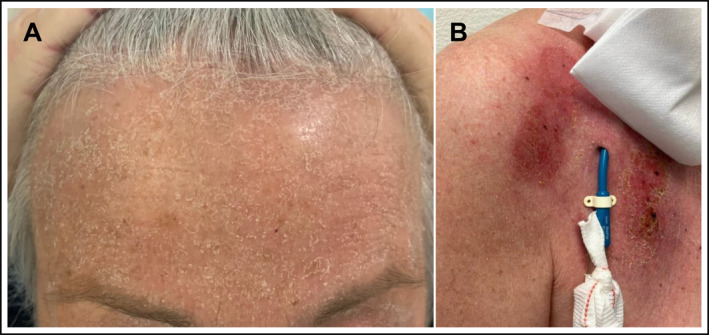
Following the administration of the fourth dose of elranatamab, the patient presented with exfoliative dermatitis and irritant contact dermatitis localized around adhesive patches and the Sheldon catheter.

Following the administration of the fourth dose of elranatamab, the patient developed exfoliative dermatitis and irritant contact dermatitis localized around adhesive patches and the Shaldon catheter (Figure 3). This reaction was likely related to severely irritated skin combined with a pre‐existing history of atopic dermatitis. The condition was effectively managed with topical therapy, including octenidindihydrochlorid, halometasonum monohydricum/triclosanum applied once daily, and urea 50 mg/macrogol‐6‐laurylether. Given the prompt resolution of symptoms, the treatment regimen was continued without dose reduction. Additionally, premedication was discontinued starting with the sixth dose, as the subsequent course remained uncomplicated.

Seven weeks after initiating elranatamab treatment, the patient achieved VGPR, evidenced by suppressed FLC and an IgG kappa paraprotein level of 2.1 g/L. (Figure 1B,C) Hemoglobin levels returned to the normal physiological range. The excellent response was confirmed in the bone marrow, approximatively 4 months after treatment initiation, with the achievement of a complete remission with flowcytometric MRD negativity (detection threshold of 10^−4^). In the absence of plasma cell infiltration, BCMA staining was not done. By fluorescence in situ hybridization (FISH), the former IGH::CCND1 fusion had become undetectable, indicating complete cytogenetic remission. Unfortunately, the patient remains dialysis‐dependent.

No secondary immunoglobulin deficiency was noted during the first 6 weeks of treatment. However, 2 months after therapy initiation, the patient had to miss the first day of cycle 3 due to an upper respiratory tract infection without an identifiable pathogen, characterized by a C‐reactive protein level of 64 mg/L and new‐onset thrombocytopenia. Given the patient's overall condition and immunosuppression, empirical antibiotic therapy with moxifloxacin was started. The thrombocytopenia was considered reactive and linked to the infection. At this time, secondary immunoglobulin deficiency was newly identified, with an IgG level of 3.1 g/L. Consequently, intravenous supplementation with polyvalent immunoglobulins was initiated. The patient rapidly recovered from the respiratory infection with full hematological regeneration. No further hematotoxicity was observed during the follow‐up, supporting the hypothesis that the thrombocytopenia was reactive and infection associated.

## 2 Role of Elranatamab in Dialysis‐Dependent RRMM

RRMM remains a significant clinical challenge, particularly in patients with renal impairment requiring dialysis. The prognosis for these patients is generally poor, with increased mortality rates [10].

Elranatamab has shown promising results in treating RRMM. The Phase 2 MagnetisMM‐3 study demonstrated an objective response rate of 61% with a complete or stringent complete response in approximately 31.7% of patients. The median time to response was 1.2 months (range: 0.9–7.4 months) [1]. These results are promising, particularly for triple‐class refractory patients previously exposed to an –imid, a proteasome inhibitor and an anti‐CD38 antibody. The European Medicines Agency (EMA) approved elranatamab in RRMM patients who have received at least three prior therapies including a proteasome inhibitor, an IMiD and an anti‐CD38 monoclonal antibody in 2023. In the Phase 1 and 2 clinical trials of teclistamab and elranatamab, renal function eligibility criteria required a minimal estimated glomerular filtration rate (eGFR) of 40 ml/min/1.73m22 and 30 ml/min/1.73m2, respectively. While the MajesTEC1‐study excluded patients with eGFR below 40 ml/min/1.73m2, the MagnetisMM‐3 study allowed inclusion of patients with more advanced renal impairment, reflecting a slightly broader eligibility criterion for renal function. However, neither trial included dialysis‐dependent patients [1, 2]. According to the local prescribing Information (Switzerland), results of population pharmacokinetic analyses indicate that mild renal impairment (60 ml/min/1.73 m2 ≤ estimated glomerular filtration rate (eGFR) < 90 ml/min/1.73 m2) or moderate renal impairment (30 ml/min/1.73 m2 ≤ eGFR < 60 ml/min/1.73 m2) did not significantly influence the pharmacokinetics of elranatamab. The pharmacokinetics and pharmacodynamics of elranatamab in the population with end stage renal disease requiring dialysis have not been extensively studied.

Van de Wyngaert et al. [4] reported the successful administration of elranatamab in a dialysis‐dependent patient with RRMM. Their patient achieved a complete response after 1 month of therapy, even though a transient pseudo‐progression was observed early in treatment, likely due to local inflammatory reactions. No unforeseen side effects were observed. There is however no evidence that the renal function improved to the point of regaining independence from dialysis.

Our case represents the second reported case in the literature of elranatamab treatment in a dialysis‐dependent patient. Elranatamab treatment was both feasible and well‐tolerated, with no unexpected severe toxicities or atypical side effects observed post‐treatment. The treatment demonstrated efficacy, evidenced by a rapid decline in free light chains after 3 weeks. The patient achieved a VGPR after two cycles, consistent with the response times reported in the literature [1]. Grade 1 CRS, as described in our case, is relatively common, with incidence rates ranging from 58% to 72% in patients receiving anti‐BCMAxCD3 bispecific antibodies [1, 2]. In our case, elranatamab was administered post‐dialysis to minimize potential drug clearance during dialysis sessions, avoid fluctuations in drug levels, and reduce the risk of interactions with dialysis‐related metabolic changes. Real‐world evidence suggests that monoclonal antibodies are not significantly cleared by dialysis and do not require dose adjustments based on renal function [10]. Monoclonal antibodies are predominantly cleared through intracellular degradation following endocytosis or phagocytosis. For multiple myeloma cases involving the t (11; 14) translocation, venetoclax is considered a viable alternative while theoretically safe in ESRD due to hepatic metabolism. However, its pharmacokinetic properties in dialysis patients remain unstudied similar to elranatamab [11]. We opted for a therapy with elranatamab for our patient, considering its safety profile, which includes manageable CRS with appropriate prophylaxis and monitoring and no significant renal toxicity. Venetoclax is associated with potential risks, including gastrointestinal adverse effects, tumor lysis syndrome, and substantial hematologic toxicities, such as neutropenia. These complications may present significant challenges in the management of dialysis‐dependent patients, who are already at high risk for complications.

Exfoliative dermatitis, a rare but severe cutaneous reaction characterized by widespread erythema, scaling, and desquamation, has been reported in patients receiving elranatamab. Cutaneous adverse events associated with elranatamab include rash and dry skin, both categorized as very common (≥ 1/10). Rash occurred in 26% of patients, but none were reported as Grade 3 or 4 in severity. Similarly, dry skin was observed in 21% of patients, with no cases reaching Grade 3 or 4 severity [1]. The skin reactions presented by our patient were mild and responded well to topical therapy. A dose modification of elranatamab was not necessary.

## 3 Other BCMA‐Directed Immunotherapies in Dialysis‐Dependent RRMM

The evidence for the use of teclistamab, a BCMA‐targeted bispecific antibody, in patients with multiple myeloma requiring dialysis is emerging, primarily from retrospective studies and case series. The use of teclistamab in this patient population has shown promising results. In a French retrospective study involving 15 dialysis‐dependent RRMM patients, teclistamab was administered subcutaneously at a dose of 1.5 mg/kg weekly following step‐up doses. The study demonstrated that teclistamab is feasible and effective in this population, with 14 out of 15 patients responding to treatment. Hemodialysis was discontinued in one patient following the recovery of renal function after 5 months of teclistamab therapy, while continuing the treatment. The responses included stringent complete responses, complete responses, very good partial responses, and partial responses. No grade 3 or 4 CRS or ICANS was reported, although infections were a significant concern, affecting 53% of patients [5]. The median time to best response was 40 days, and the median progression‐free survival had not been reached at a median follow‐up of 5.4 months.

In another case series, four heavily pretreated myeloma patients on hemodialysis in the United States were treated with teclistamab. The patients tolerated the therapy well, with only one patient experiencing grade 1 CRS, managed with supportive care with no evidence that any patient in the cohort became dialysis‐independent [6]. The pharmacokinetics of teclistamab seem not significantly impacted by dialysis due to its large molecular weight and elimination primarily through intracellular catabolism rather than renal clearance. These findings indicate that teclistamab is a viable treatment option for RRMM patients on dialysis, offering a potential therapeutic option for this challenging patient subgroup [12].

Belantamab mafodotin, a BCMA‐targeting immunoconjugate not renally metabolized, has been evaluated in patients with severely impaired renal function, including those requiring dialysis. A phase I study assessed its pharmacokinetics, safety, and efficacy in this patient population, showing it to be generally well‐tolerated and effective, though dose adjustments may be needed due to altered pharmacokinetics [13]. A post‐hoc analysis of the DREAMM‐2 trial examined outcomes in RRMM patients with varying renal function, finding similar rates of keratopathy and albuminuria but higher frequencies of anemia, pyrexia, and thrombocytopenia in those with impairment. Most patients showed stable or improved eGFR [14]. However, data on dialysis patients remain limited.

Chimeric Antigen Receptor (CAR) T‐cell therapy, specifically idecabtagene vicleucel (ide‐cel), has shown efficacy in treating relapsed/refractory multiple myeloma (RRMM) [15, 16]. The KarMMa trial excluded patients with renal impairment (creatinine clearance [CrCl] < 45 mL/min, Cockcroft‐Gault equation) [17]. A retrospective study by Sidana et al. found comparable response rates (93% vs. 82%) and survival outcomes in RRMM patients receiving idecabtagene vicleucel with renal impairment (CrCl < 50 mL/min. including ESRD), though higher rates of short‐term high‐grade cytopenias were observed. By day 60 post‐therapy, 73% of patients with CrCl < 30 mL/min had grade III or higher thrombocytopenia, with 36% experiencing persistent grade III or higher anemia and neutropenia. Renal function remained stable post‐therapy [18]. A case report described the successful idecabtagene vicleucel administration in a dialysis‐dependent patient using reduced fludarabine dosing and hemodialysis, achieving a complete remission lasting beyond 12 months.

## 4 Additional Considerations for the Administration of Elranatamab and Teclistamab in Patients With ESRD

The use of BCMA‐targeted bispecific antibodies in the treatment of RRMM has been associated with a significant risk of infections, primarily due to profound hypogammaglobulinemia and T‐cell depletion. Infections are a major concern, with studies showing that up to 56% of patients experience all‐grade infections, and about 24% experience grade 3 or higher infections [19]. The risk is particularly high during periods of disease remission, likely due to the immunosuppressive effects of the therapy. Unfortunately, our patient experienced a viral upper respiratory tract infection due to the rapid onset of secondary immunoglobulin deficiency, approximately 8 weeks after treatment initiation despite full vaccination and adherence to the suggested infectious prophylaxis. Management strategies should include vigilant infection screening and prophylaxis. Prophylactic intravenous immunoglobulin has been shown to reduce the incidence of severe infections by up to 90%, highlighting its importance in managing hypogammaglobulinemia. Early initiation of supplementation with polyvalent immunoglobulins during the initial months of treatment is recommended to mitigate this risk. Additionally, routine prophylaxis against common viral infections, such as varicella‐zoster virus (VZV) and a PcP prophylaxis, is advised, along with regular monitoring for reactivation of latent infections. In patients receiving BCMA‐targeted bispecific antibodies, vaccination against influenza and COVID‐19 is also recommended as part of infection prevention strategies. Vaccination should ideally occur before the initiation of bispecific antibody therapy to maximize immune response, as antibody production may be impaired once treatment has started [20]. Unlike one‐time therapies like CAR‐T‐cells, bispecific antibodies require ongoing administration, which prolongs immunosuppression and increases the risk of infection over time. In the MajesTEC‐1 study on teclistamab, switching from weekly to biweekly dosing significantly reduced grade ≥ 3 infections from 33.3% to 15.6% [21]. The rationale for spacing injections lies in allowing partial recovery of immune function between doses. Spacing injections is a highly viable approach for managing infection risks associated with bispecific antibodies, especially for patients achieving sustained responses. This strategy should be tailored to individual patient responses and balanced against disease control needs. These measures are crucial for minimizing infection‐related morbidity and mortality in this vulnerable patient population.

A large registry‐based study reported a renal recovery rate of 9.1% among multiple myeloma (MM) patients on chronic dialysis [22]. BCMA‐directed treatments, by rapidly reducing plasma cell burden and free light chain production and achieving a deep hematologic response, may contribute to renal recovery if initiated early enough before irreversible damage occurs [23]. In dialysis‐dependent MM patients who fail to achieve a complete response with first‐line therapies such as daratumumab combined with bortezomib, lenalidomide, and dexamethasone, the decision to early transition to a potent agent like elranatamab warrants careful consideration. Given elranatamab's efficacy and its potential to induce profound hematologic responses, an early switch to this agent could hypothetically enhance the probability of achieving complete remission early and improve renal function. This hypothesis is supported by experiences such as those noted in our patient, where early administration of elranatamab may have contributed to dialysis independence. These findings underscore the need for further investigation through prospective studies or clinical trials.

## 5 Conclusion

RRMM in patients with ESRD requiring hemodialysis poses a significant clinical challenge due to poor prognosis and limited therapeutic options. While elranatamab and teclistamab have emerged as promising therapies for RRMM, their efficacy and safety in dialysis‐dependent patients are not well established [1, 2].

To the best of our knowledge, this report represents the second case in the literature indicating that elranatamab may be an effective and feasible treatment option for RRMM in patients with renal impairment requiring hemodialysis. These findings and data from the literature support the potential use of bispecific antibodies in ESRD patients, provided that careful monitoring and robust infection prevention strategies are implemented. By highlighting a second case of elranatamab in a dialysis‐dependent RRMM patient and synthesizing the literature, we aim to provide valuable insights for clinicians managing such challenging patient cases. We hope that this literature overview will serve as a practical resource to guide treatment decisions and improve outcomes in this vulnerable population. Prospective trials with larger cohorts and extended follow‐up are needed to validate this hypothesis and to optimize treatment scheduling and dosing in this demanding myeloma patient population.

## Consent

The patient has provided informed consent for the publication of this case report.

## Conflicts of Interest

The authors declare no conflicts of interest.

## Peer Review

The peer review history for this article is available at https://www.webofscience.com/api/gateway/wos/peer-review/10.1002/hon.70120.

## Data Availability

The data that support the findings of this study are available from the corresponding author upon reasonable request.
